# Life history and population ecology of *Radix swinhoei* (Lymnaeidae) in nearshore regions of a hypereutrophic plateau lake

**DOI:** 10.1002/ece3.9631

**Published:** 2022-12-14

**Authors:** Junqian Zhang, Zhuoyan Song, Zhengfei Li, Jiali Yang, Zhicai Xie

**Affiliations:** ^1^ State Key Laboratory of Freshwater Ecology and Biotechnology, Institute of Hydrobiology Chinese Academy of Sciences Wuhan China; ^2^ Great Lakes Institute for Environmental Research University of Windsor Windsor Ontario Canada; ^3^ University of Chinese Academy of Sciences Beijing China

**Keywords:** eutrophication, Pulmonata, secondary production, size structure

## Abstract

Accurate assessment of life history and population ecology of widespread species in ultra‐eutrophic freshwater lakes is a prerequisite for understanding the mechanisms by which widespread species respond to eutrophication. Freshwater pulmonate (*Radix swinhoei*) is widespread and abundant in many eutrophic water bodies in Asia. Despite its key roles in eutrophic lake systems, the information on life history and population ecology of *R. swinhoei* is lacking, especially in ultra‐eutrophic freshwater plateau lakes. Here, we conducted a 1‐year survey of *R. swinhoei* with monthly collections to measure the life history traits (life span and growth), annual secondary production, and population size structure of *R. swinhoei* in nearshore regions with a high seasonally variation of nutrients in Lake Dianchi, a typic hypereutrophic plateau lake in Southwest China. Our results showed that *R. swinhoei* had the highest biomass in autumn and had the lowest in winter. Its maximum potential life span was 2.5 years, with three recruitment periods (November, March, and July) within a year. Its annual secondary production and P/B ratio were 137.19 g WW/m^2^ and 16.05, respectively. Redundancy analysis showed that eutrophication‐related environmental factors had weak correlations with population size structure of *R. swinhoei*. Our results suggested that *R. swinhoei* is a typical r‐strategist with high secondary production and thrive in eutrophic environment. Our study can help better understand the mechanisms for widespread species to survive eutrophication and could also be relevant for biodiversity conservation and management of eutrophic ecosystems.

## Graphical abstract

The life history traits (life span and growth), annual secondary production, and population size structure of Asia widespread pulmonate *Radix swinhoei* were carefully and systematically examined across 1 year in nearshore regions of a hypereutrophic plateau lake, Lake Dianchi. The maximum potential life span of *R. swinhoei* was 2.4 years, with three recruitment periods (November, March, and July) within a year. *Radix swinhoei* was a typical r‐strategist with high secondary production and P/B ratio, but showed weak correlations between its population size structure and eutrophication‐related environmental factors, suggesting this snail can thrive in eutrophic conditions.

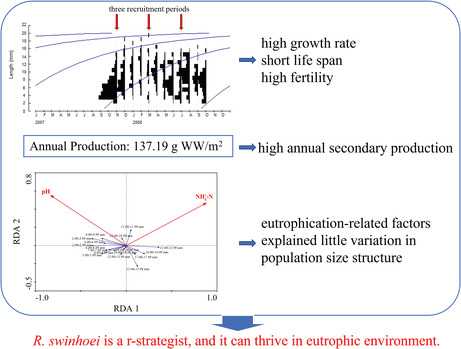

## 1 INTRODUCTION

Understanding how species respond to environmental changes is highly relevant to biodiversity conservation and ecosystem management (Martínez‐Blancas et al., 2018; Siqueira et al., 2012). Widespread species are expected to have a wider niche breadth and may utilize a wider variety of resources than rare species (Slatyer et al., 2013). One of the potential mechanisms for widespread species to thrive across a broad range of environments is related to their ecological traits (Slatyer et al., 2013). For instance, widespread species can disperse to keep track of preferential habitats, or they may grow fast with a short maximum potential longevity and high fecundity to maintain viable populations in unfavorable environmental conditions (Brown, 1984; Kunin & Gaston, 1993; Kunin & Shmida, 1997; Slatyer et al., 2013; Vincent et al., 2020). In freshwater ecosystems, these r‐selected life history traits can enable widespread species to survive eutrophication (Römermann et al., 2008; Zhang et al., 2020) and may even allow them to replace rare (endemic) species that have a low tolerance to algal toxins and anoxic condition, thus resulting in biodiversity loss, biotic homogenization and alteration of energy flow through trophic levels in eutrophic ecosystems (Hillebrand et al., 2008; Leprieur et al., 2008; McKinney & Lockwood, 1999; Olden et al., 2011; Toussaint et al., 2016). Despite their potential roles in freshwater ecosystems, the information on life history of widespread species in eutrophic systems and their ecological response to eutrophication is still lacking, especially in ultra‐eutrophic freshwater plateau lakes. Such information is valuable because it could aid in biodiversity protection in eutrophic freshwater ecosystems.

**FIGURE 1:**
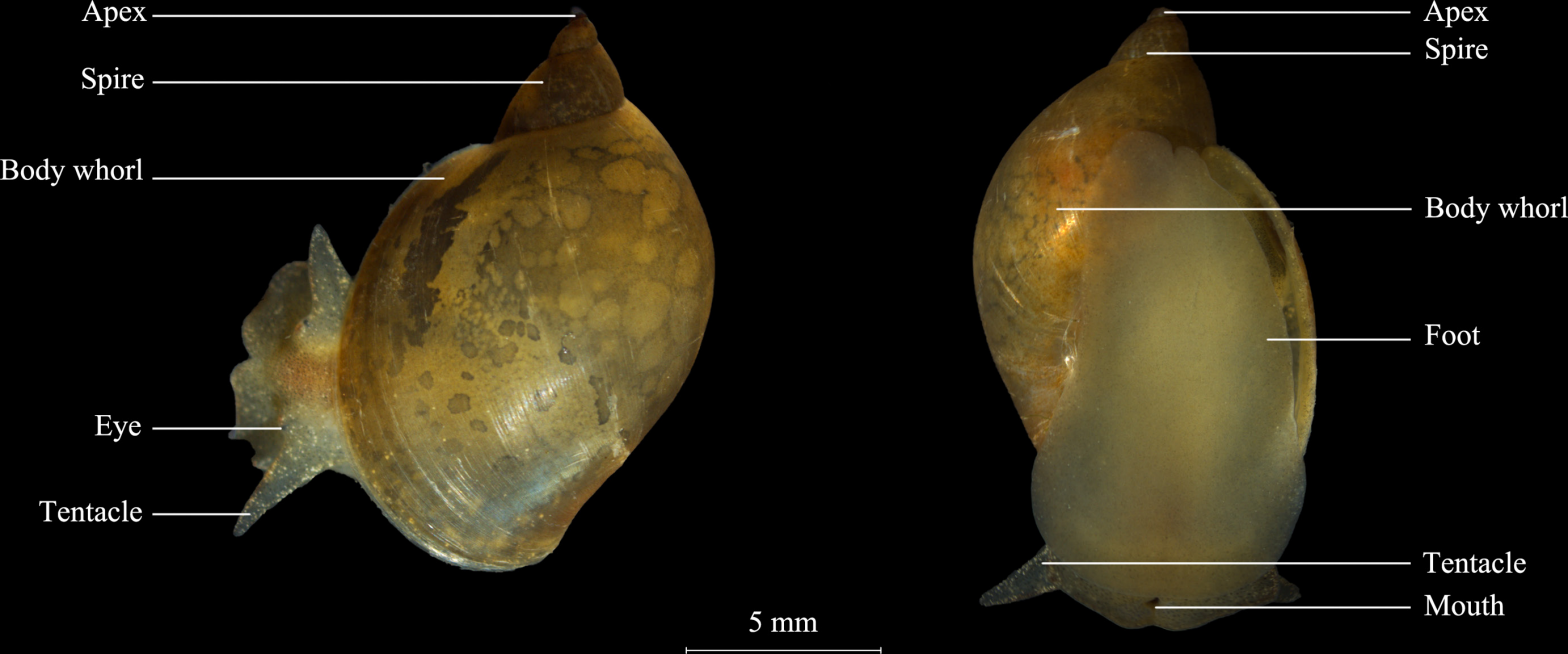
Photograph of *Radix swinhoei*

*Radix swinhoei* (Adams, 1866), a typical freshwater pulmonate (Figure 1; Zhang et al., 2012), inhabits a wide variety of slow‐flow ecosystems across Asia, including streams, ponds, rice paddies, canals, and nearshore regions of lakes (Liu et al., 1979). This snail is oviparous, hermaphroditic and tends to live on aquatic plant regions in shallow lentic environments, where they can graze epiphytic organisms and plant matter (Liu et al., 1979). *R. swinhoei* has a vascularized lung in its mantle cavity which can keep air to regulate its buoyancy to glide up and down in water (Barnes, 1987; Brown & Lydeard, 2010), making them equipped with stronger dispersal ability compared to prosobranch snails living on the bottom sediment. As a widespread mollusk in Asia, *R. swinhoei* could promote growth of aquatic plants by removing the periphyton from surface of the plants through grazing to reduce nutrient and light competitions (Jones et al., 1999; Underwood, 1991; Underwood et al., 1992; Zhi et al., 2020). *R. swinhoei* and submerged plants can control cyanobacteria blooms synergistically, which offers a promising approach for eutrophication control (Carpenter & Lodge, 1986; Li et al., 2009; Zhang et al., 2015). As one of the main food sources for higher predators such as fish and aquatic birds, *R. swinhoei* can constitute a substantial proportion of overall secondary production in nearshore regions of lakes (Zhang et al., 2012).

In addition, this species can tolerate nutrient pollution to some extent in water bodies including lakes (Zhang et al., 2016). In Lake Dianchi, for example, *R. swinhoei* were frequently found on shore and on aquatic plants in the nearshore regions of Lake Dianchi (Zhang et al., 2015), even after decades of eutrophication and frequent cyanobacteria blooms in this plateau lake, which has caused dramatic local biodiversity loss and population decline of local species such as the endemic gastropod *Margarya melanioides* (Song et al., 2013, 2017; Ye et al., 2015; Zhang et al., 2020). Why *R. swinhoei* survives eutrophication remain unclear, which emphasizes the need to better understand its life history, secondary production, and population ecology in eutrophic lakes. This information is important and could provide insight on the management of biodiversity and eutrophication in Lake Dianchi and other relevant freshwater ecosystems.

In this study, we focused on life history, secondary production, and population ecology of *R. swinhoei* in nearshore regions of a eutrophic lake. We predicted that (1) *R. swinhoei* would be a r‐strategist with high secondary production and short life span; (2) population size structure of *R. swinhoei* would show weak correlations with eutrophication‐related environmental factors because they can survive eutrophication. To test these two predictions, we conducted a field investigation in nearshore regions of Lake Dianchi across 1 year with 12 collections. Within the one‐year monthly study in a research area where the nutrients varied greatly in different months, we aim to (1) quantify population dynamic and growth as well as secondary production of *R. swinhoei* and (2) explore relationships between eutrophication‐related environmental factors and population size structure of *R. swinhoei*.

## 2 MATERIALS AND METHODS

### 2.1 Study area

Lake Dianchi (24°23′–26°22′N, 102°10′–103°40′E), the sixth‐largest freshwater lake in China, lies in a plateau city of Kunming, Yunnan Province, with a characteristic tropical plateau monsoon climate and hydrological conditions. It is one of the three most seriously polluted large shallow freshwater lakes in China (other two are Chaohu and Taihu; Zhang et al., 2012).

**FIGURE 2:**
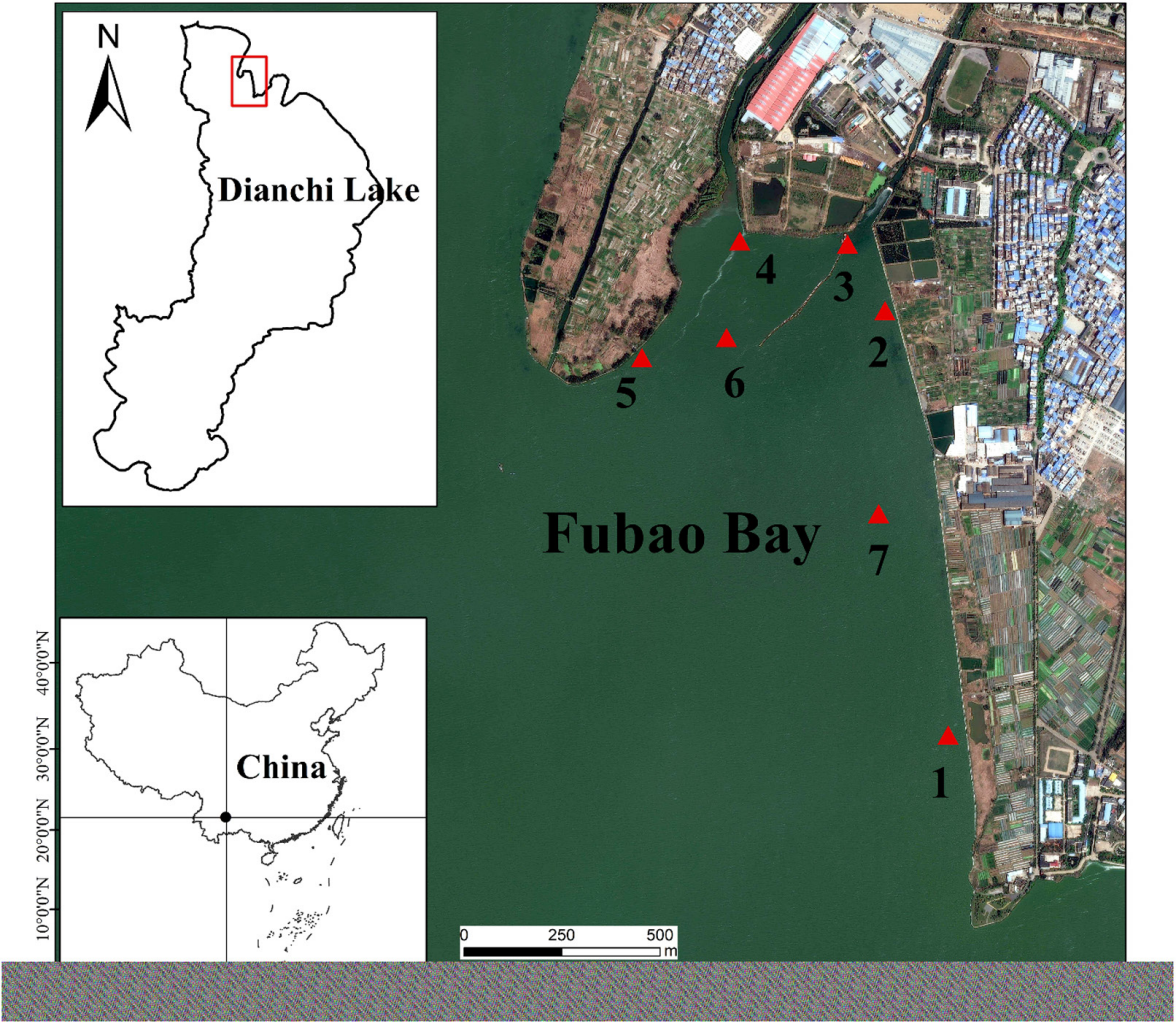
Sampling sites of *Radix swinhoei* in Fubao Bay of Lake Dianchi

The study was performed in the Fubao Bay (24°25′N, 102°41′E; Figure 2), a nearshore region in the northeast of Lake Dianchi. We selected Fubao Bay as the study area because it has been seriously polluted with large spatiotemporal variation in nutrients and algal biomass (cyanobacteria blooms tend to accumulate in summer and rarely occur in winter), which provides opportunity for studying life history of *R. swinhoei* and its ecological response to eutrophication.

### 2.2 Sampling and laboratory work

We sampled *R. swinhoei* monthly from November 2007 to October 2008 at seven sampling sites in the Fubao Bay, with Site 1–5 near shoreline and Site 6 and 7 in aquatic plant zones (Figure 2). This period is considered to be the most serious period of eutrophication and algae bloom in Dianchi Lake in recent decades (Liu et al., 2021; Wang et al., 2019). Since *R. swinhoei* prefers near shoreline zones and aquatic habitats, our sampling was mainly carried out in these two habitats. We used a 20 × 25 cm quantitative frame to collect snail samples. We fixed two areas with the quantitative frame at each sample site. For the near shoreline shallow sites (Site 1–5), we shoveled the surface sediments and snail individuals within the areas of the quantitative frame into the sieve (sieve mesh: 500 μm). For the aquatic plant zones (Site 6 and 7), we harvested all the aquatic plants within the areas of the quantitative frame into the sieve (sieve mesh: 500 μm). Then, we carefully washed the sediments or aquatic plants in the sieve and bagged the samples. At each site, two samples were mixed. All samples were taken back to the laboratory, and all individuals of *R. swinhoei* were manually picked out. All collected *R. swinhoei* were preserved in 3%–5% formaldehyde solution in field and taken back to laboratory for further analysis.

In the laboratory, we counted and weighted all collected *R. swinhoei*. Before the weight measurement, sampled *R. swinhoei* was dried with neutral filter paper and then weighed with an electronic balance. We then measured shell length (*L*) with a vernier caliper to the nearest 0.02 mm for each of the individuals with *L* ≥ 5 mm. For those with *L* < 5 mm, we measured their *L* under a dissecting microscope with an ocular micrometer to the nearest 0.01 mm.

Water temperature (T), pH, and dissolved oxygen (DO) were measured using a YSI meter in situ. Meanwhile, the surface water was collected from one depth (within 0.5 m) using 1‐L bottle at each of the seven sampling sites and then transported on ice in a cooler to the laboratory for the analysis of water quality parameters including total phosphorus (TP), total nitrogen (TN), ammonia–N (${\mathrm{NH}}_{4}^{+}$–N), chemical oxygen demand (permanganate index, COD_Mn_), and chlorophyll *a* (Chl‐*a*) according to the Chinese Water Analysis Methods Standards (Huang et al., 1999).

### 2.3 Data analysis

The relationship between shell length (*L*) and body wet weight (WW) was quantified based on the following equation:

$$\mathrm{WW}=a\times L^{b},\text{or}\;\ln\;\mathrm{WW}=\ln\;a+b\;\ln\;L$$

where ln *a* and *b* are intercept and slope of the *L*–WW relationship. *B* is considered to be around 3.0 (Benke & Huryn, 2006; Konstantinov, 1958).

All collected snails were sorted into 18 size groups at 1 mm intervals starting from 2 mm. In fact, a total of 1613 individuals were collected, with the following monthly numbers (from November 2007 to October 2008): 698, 131, 49, 31, 34, 69, 93, 104, 105, 110, 77, 112. Data analysis was based on calculated density data. The growth parameters (see details below) were estimated based on body length frequency distribution by fitting the following von Bertalanffy growth equation:

$$L_{t}=L_{\infty }\left[1-e^{-K\left(t-t_{0}\right)}\right]$$

where *L*_*t*_ is the average length at time t, *L*_*∞*_ is the theoretical asymptotic length and *K* is the population growth rate, which was estimated through ELEFAN I program in FiSAT‐II software. ELEFAN I program is commonly used in gastropod study (Song et al., 2017; Vasconcelos et al., 2006). *T*_*0*_ is the theoretical age of gastropods at length zero. It is generally a negative value, but does not mean “prenatal growth” (Gayanilo et al., 2005). The equation of *t*_*0*_ is as follows (Moreau, 1987):

$$t_{0}=1/K\times \ln\left[\left(L_{\infty }-L_{h}\right)/L_{\infty }\right]$$

where *L*_*h*_ is the theoretical hatching length (i.e., the length at *t* = 0), which corresponds to the minimum length of the sampled juveniles (2.17 mm; Simão & Soares‐Gomes, 2017).

The maximum potential longevity is estimated using the following equation (Doinsing & Ransangan, 2022; Pauly & Munro, 1984; Song et al., 2017):

$$t_{\max}=3/K$$

Secondary production of *R. swinhoei* was quantified using the size‐frequency method. We used the size‐frequency method because it is hard to effectively identify cohorts of *R. swinhoei* and this method does not require tracking cohorts (Benke, 1979; Benke & Huryn, 2006, 2017; Hamilton, 1969). The equation of calculating secondary production is as follows:

$$P=a\left[\sum_{i=1}^{a-1}\frac{\left(W_{i}+W_{i+1}\right)}{2}\times \left(N_{i}-N_{i+1}\right)+W_{a}\times N_{a}\right]\times \frac{12}{\mathrm{CPI}}$$

In the equation above, *P* is the production, *a* is the number of shell‐length groups, *I* and *i + 1* are the two consecutive shell length groups, *W* is average individual weight, *N* is the density, *W*_*a*_ is the average individual weight of the last group (i.e., group *a*), *N*_*a*_ is the density of the last group (i.e., group *a*), CPI (i.e., cohort production interval) means the average time of development (in days) from hatching to the production of the first brood. Here, the CPI is measured in months (Benke, 1979; Benke & Huryn, 2006).

Multivariate analyses were conducted in R environment 4.0.1 (R Development Core Team, 2020). Detrended correspondence analysis (DCA) was performed to determine the appropriate type of model for direct gradient analysis (Ter Braak & Verdonschot, 1995), which indicated that a linear model (gradient lengths < 3 standard units) would best fit the data. Then, redundancy analysis (RDA) was selected to evaluate the influence of environmental variables on the size structure of *R. swinhoei*. Before RDA analysis, logarithmic transformation was performed on age structure data and environmental variables which did not' conform to normality assumption (Shapiro–Wilk test, *p* < .05). The environmental variables that had variance inflation factors >20 were removed from the analysis to avoid high collinearity. The significance of the RDA full model has been tested before the variable selection (“ANOVA” R function). Only if the full model was significant, forward selection was conducted to select a parsimonious set of explanatory variables under the cutoff point of 0.05 (“ordiR2step” R function). The explanatory power of the final RDA models was obtained by calculating the adjusted *r* ^2^ values (“RsquareAdj” R function), and these are unbiased as noted by Peres‐Neto et al. (2006).

## 3 RESULTS

### 3.1 Physical and chemical parameters

The average concentrations of TN, TP, COD_Mn_, and Chl‐*a* in the sampling area were 10.41, 1.91, 52.67, and 0.59 mg/L (Table 1), respectively. Particularly, sites 2 and 3 had mean TN, TP, COD_Mn,_ and Chl‐a higher than the other four sites (Table 1). The high standard deviation of each physical and chemical factor indicated that the environmental factors at each sampling sites changed greatly during the sampling period (Table 1).

**TABLE 1:** Mean (± standard deviation) values, maximum values (max), and minimum values (min) of water quality parameters averaged across 12 months at seven sampling sites in the Fubao Bay, Lake Dianchi

Site	1	2	3	4	5	6	7	Mean
pH								
Mean	9.41 ± 0.83	8.84 ± 0.81	8.51 ± 0.75	8.99 ± 0.73	9.26 ± 0.82	9.26 ± 0.82	9.43 ± 0.94	9.11 ± 0.84
Max	10.31	10.16	10.01	9.93	10.41	10.45	10.61	
Min	8.42	7.91	7.95	7.62	8.16	8.21	8.04	
DO (mg/L)								
Mean	8.6 ± 3.8	5.1 ± 2.6	3.9 ± 2.3	6.5 ± 2.1	7.86 ± 2.72	7.92 ± 2.81	7.28 ± 2.53	6.81 ± 3.04
Max	13.70	9.05	8.44	9.95	11.25	11.26	10.61	
Min	5.00	0.26	2.10	0.27	4.81	3.25	8.04	
*T* (°C)								
Mean	19.54 ± 5.64	19.28 ± 5.75	19.91 ± 5.91	19.29 ± 5.70	19.35 ± 5.84	19.22 ± 6.32	18.89 ± 5.48	19.31 ± 5.51
Max	26.30	25.50	25.80	25.60	25.30	26.40	23.90	
Min	11.00	11.00	11.00	11.20	10.90	10.80	11.30	
TN (mg/L)								
Mean	4.23 ± 2.41	19.1 ± 43.7	25.9 ± 39.5	6.11 ± 3.22	7.42 ± 4.51	7.80 ± 6.53	3.71 ± 2.36	10.41 ± 22.57
Max	7.32	143.6	114.15	13.04	12.67	21.6	8.82	
Min	0.43	2.37	4.52	2.55	2	2.2	0.51	
TP (mg/L)								
Mean	0.33 ± 0.16	4.60 ± 12.92	6.37 ± 11.3	0.68 ± 0.47	0.74 ± 0.49	0.80 ± 0.79	0.28 ± 0.18	1.91 ± 6.53
Max	0.63	41.4	30.61	1.87	1.49	2.8	0.72	
Min	0.11	0.24	0.35	0.29	0.23	0.25	0.14	
${\mathrm{NH}}_{4}^{+}$–N (mg/L)								
Mean	0.81 ± 1.17	2.03 ± 1.83	2.09 ± 2.86	1.19 ± 1.56	1.06 ± 1.46	0.80 ± 1.16	0.45 ± 0.52	1.19 ± 1.66
Max	2.14	4.45	5.22	9.41	4.39	3.87	1.60	
Min	0.02	0.08	0.17	0.06	0.02	0.02	0.02	
COD_Mn_ (mg/L)								
Mean	17.5 ± 7.5	214.2 ± 413.4	155.50 ± 230.5	21.78 ± 11.49	25.61 ± 14.73	26.68 ± 18.40	14.30 ± 5.90	52.67 ± 159.97
Max	72.65	1180.39	579.20	39.98	64.16	99.16	38.07	
Min	5.82	3.93	7.56	6.00	7.65	6.00	7.10	
Chl*‐a* (mg/L)								
Mean	0.21 ± 0.24	0.34 ± 0.58	2.36 ± 6.28	0.28 ± 0.25	0.46 ± 0.42	0.45 ± 0.40	0.17 ± 0.13	0.59 ± 2.29
Max	0.83	1.97	19.13	0.88	1.18	2.21	0.43	
Min	0.03	0.04	0.05	0.05	0.04	0.04	0.03	

### 3.2 Spatiotemporal variation of population density and biomass

**FIGURE 3:**
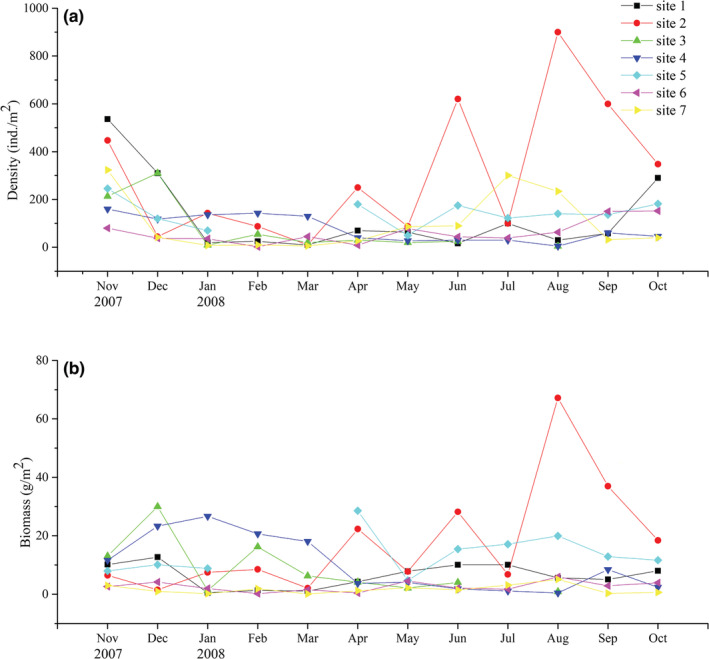
Temporal and spatial dynamics of the density (a) and biomass (b) of *Radix swinhoei* in Fubao Bay, Lake Dianchi

Figure 3 showed the spatiotemporal variations of population density and biomass of *R. swinhoei* in Fubao Bay. From November 2007 to October 2008, average density of *R. swinhoei* in the Fubao Bay was highest in November 2007 (286.5 ind./m^2^), and decreased sharply from December 2007 to March 2008 (36.8 ind./m^2^), and then rose to 196.7 ind./m^2^ in August 2008. The mean *R. swinhoei* biomass started to decrease from December 2007 (11.82 g/m^2^) and was at a lower level in March and May, and then increased up to in August 2008 (15.01 g/m^2^). The annual mean density and biomass were 125.2 ind./m^2^ and 8.54 g/m^2^, respectively. The highest average density and biomass occurred at sampling site 2 (303.1 ind./m^2^ and 17.79 g/m^2^); the lowest annual average density and biomass were at site 3 (57.2 ind./m^2^) and at site 7 (1.67 g/m^2^), respectively.

### 3.3 Life history traits and secondary production

**FIGURE 4:**
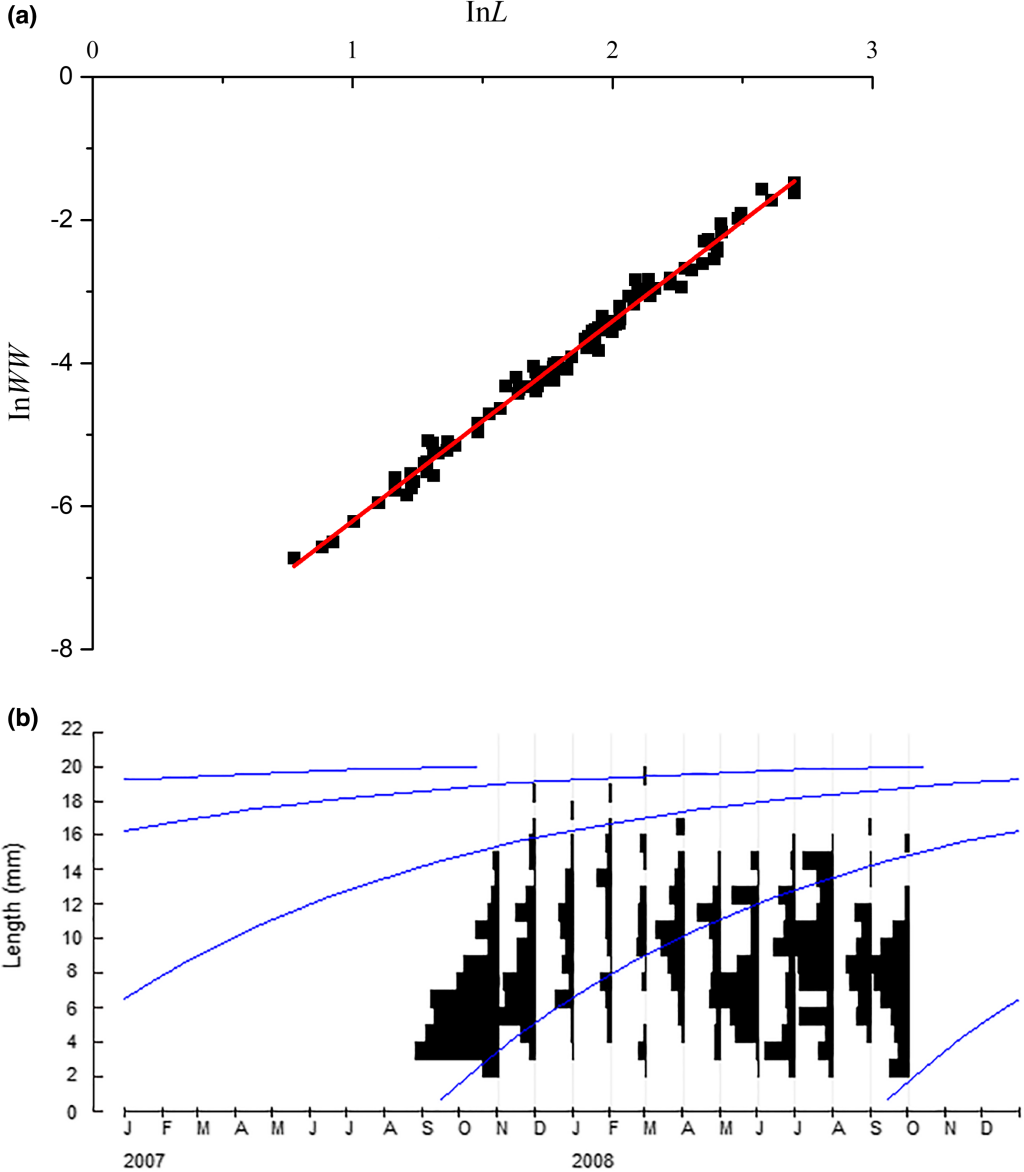
Life history traits of *Radix swinhoei* in the Fubao Bay, Dianchi Lake. (a) Relationship between shell length (*L*) and body weight (*WW*); (b) length‐frequency histogram of *R. swinhoei* from November 2007 to October 2008, with shell growth curves (*L*_*∞*_ = 20.48 mm, *K* = 1.2 year^−1^, *t*_0_ = −0.093 years).

There was a linear relationship between the natural logarithm of shell length (*L*) and the natural logarithm of body weight (wet weight, WW) of *R. swinhoei* (Figure 4a). The regression equation was as follows:

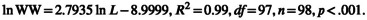




Based on shell length frequency of *R. swinhoei*, the growth curve with the highest score of 0.258 (obtained through the ELEFAN I program) was selected. The theoretical asymptotic length (*L*_*∞*_) and the population growth rate (*K*) were estimated to be 20.48 mm and 1.2 year^−1^, respectively.

The von Bertalanffy growth equation is shown in the following formula:

$$L_{t}=20.48\left[1-e^{-1.2\left(t+0.093\right)}\right]$$

The minimum sampled juvenile length (*L*_*h*_) was 2.17 mm. The initial theoretical age at length zero (*t*_*0*_) was −0.093 years, and the potential life span was 2.50 years (i.e., 912 days).

Recruitment of *R. swinhoei* occurred in spring, summer, and fall in the Lake Dianchi, with three peaks in November, March, and June–August (Figure 4b). There were three cohorts of *R. swinhoei* that appeared in 1 year, with some overlap in the body frequency distribution.

The average value of CPI was about 4 months. The annual secondary production (wet weight) was 137.19 g WW/m^2^, and the annual P/B coefficient was 16.05 (Table 2).

**TABLE 2:** Calculation of annual production of *Radix swinhoei* using the size‐frequency method

Size class (mm)	Density^a^ (No./m^2^)	Biomass (g/m^2^)	Average individual weight (g)	No. lost (No./m^2^)	Mass at loss (mg)	Biomass lost (g/m^2^)	Times No. size classes
2.00–2.99	2.68	0.004	0.0014	−6.41	0.003	−0.017	−0.30^b^
3.00‐3.99	9.09	0.035	0.0039	−0.28	0.006	−0.002	−0.03^b^
4.00–4.99	9.38	0.081	0.0087	−3.80	0.012	−0.045	−0.81^b^
5.00–5.99	13.18	0.197	0.0150	−0.70	0.019	−0.013	−0.24^b^
6.00–6.99	13.88	0.323	0.0233	−1.88	0.030	−0.056	−1.01^b^
7.00–7.99	15.76	0.579	0.0368	1.71	0.045	0.076	1.37
8.00–8.99	14.05	0.737	0.0525	2.58	0.062	0.160	2.89
9.00–9.99	11.47	0.822	0.0717	0.65	0.082	0.053	0.95
10.00–10.99	10.83	1.004	0.0927	2.09	0.109	0.228	4.10
11.00–11.99	8.74	1.096	0.1255	2.46	0.145	0.358	6.45
12.00–12.99	6.27	1.037	0.1654	3.06	0.187	0.572	10.30
13.00–13.99	3.22	0.673	0.2092	−0.67	0.225	−0.150	−2.70
14.00–14.99	3.88	0.935	0.2408	2.45	0.277	0.679	12.23
15.00–15.99	1.43	0.450	0.3138	0.61	0.336	0.203	3.66
16.00–16.99	0.83	0.297	0.3579	0.72	0.402	0.290	5.22
17.00–17.99	0.11	0.048	0.4461	−0.14	0.506	−0.069	−1.25
18.00–18.99	0.25	0.139	0.5668	0.11	0.568	0.060	1.09
19.00–19.99	0.14	0.079	0.5692	0.14^c^	0.569^d^	0.079^d^	1.42
Biomass (*B*)		=8.54 g/m^2^		Production (uncorrected) (*P*)		=45.73 g WW/m^2^	
Cohort *P*/*B* ^e^		=5.35		Annual *P* (Prod. *12/4)		=137.19 WW/m^2^	
Annual *P*/*B*		=16.05					

*Note*: Density (No./m^2^), *N*; biomass (g/m^2^), B=N×W; average individual weight (g), $W_{i}=B_{i}/N_{i}$; No. lost (No./m^2^), $∆N=N_{i}-N_{i+1}$; mass at loss (mg), $\bar{W}=\left(W_{i}+W_{i+1}\right)/2$; biomass lost (g/m^2^), $∆B=\bar{W}∆N$; times No. size classes, $∆P=\bar{W}∆N\times 18$; production (uncorrected), $P=\sum_{1}^{18}∆P$; annual *P*, annual production, $\text{Annual}\;P=P\left(\text{uncorrected}\right)\times \frac{12}{\mathrm{CPI}}$, the average value of CPI was about 4 months.

^**a**^ Density (No./m^2^) column is the average value from samples taken throughout 1 year for each size class.

^b^ Negative value at top of table (right column) disregarded since it is probably an artifact caused by inefficient sampling of smallest size class or rapid growth through size interval. If negative values are found below a positive value, they should be included in the summation (Benke & Huryn, 2017).

^c^ The last “No. Lost” value should be equal to density of the largest size class.

^d^ The last “Mass at Loss” value should be equal to average individual weight of the largest size class.

^e^ *P*/*B* (Production divided by biomass) is the weighted average of the biomass growth rate of all individuals in a population. Cohort *P*/*B* is defined as the production of a population over its life span divided by the average biomass over the same period. The property of cohort *P*/*B* is that it has a relatively constant value of about 5 (the range is usually 3–8; Benke & Huryn, 2017).

### 3.4 Relationships between population size structure and environmental variables

**FIGURE 5:**
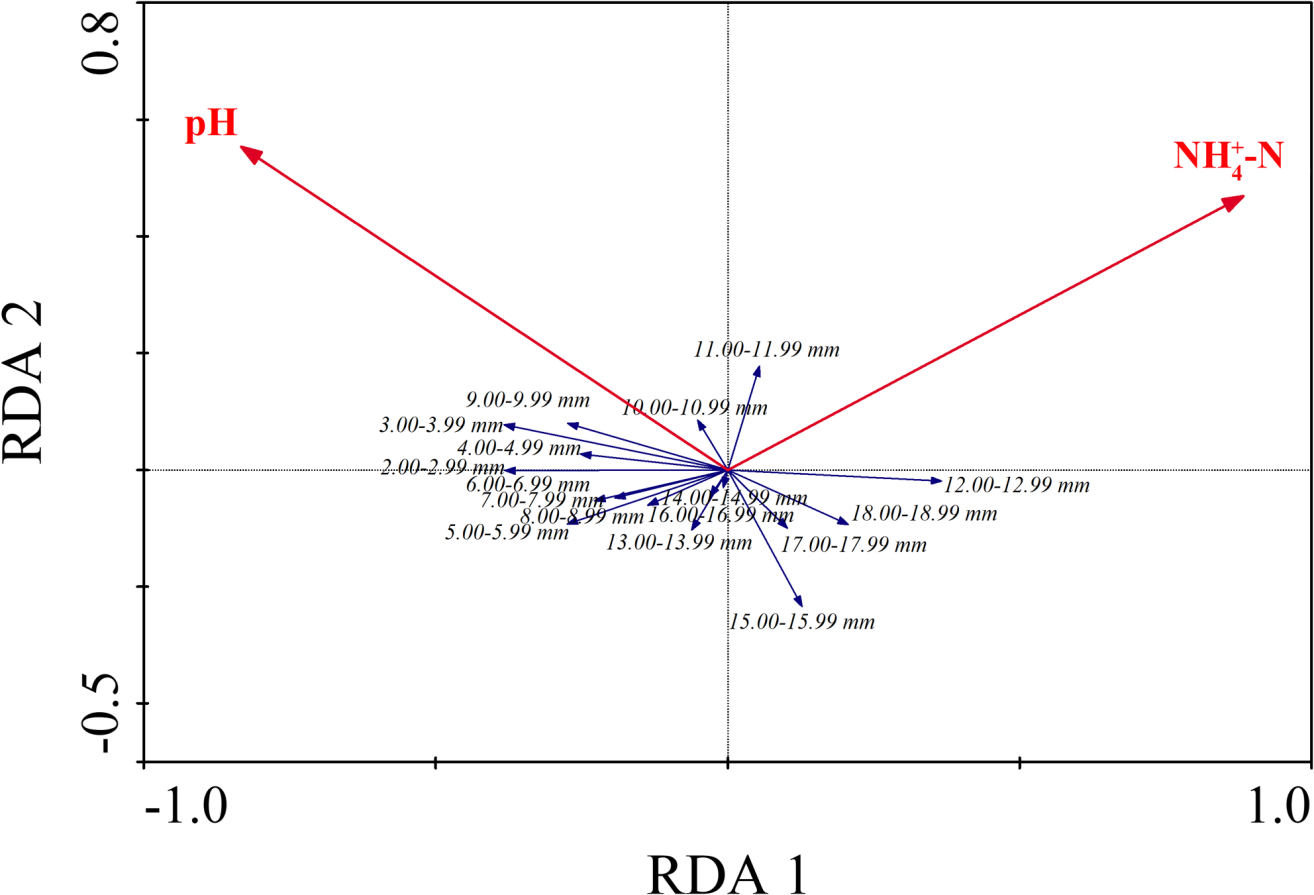
Redundancy analysis plot showing the relationship between water quality variables and the age structure composition of *Radix swinhoei* in Fubao Bay of Lake Dianchi. Arrow length and angles between arrows represent the strength of correlations between water quality variables and size classes.

The RDA analysis showed that ${\mathrm{NH}}_{4}^{+}$–N and pH were the key environmental factors for the age structure composition of *R. swinhoei* (Table 3, Figure 5). However, these two environmental variables only accounted for 3.3% of the age structure composition of *R. swinhoei* in our study area (Table 3).

**TABLE 3:** Redundancy analysis results showing the relative influence of significant environmental variables on the age structure composition of *Radix swinhoei* in Fubao Bay, Lake Dianchi.

Water quality variable	Accumulative *R* ^2^	Accumulative adj. *R* ^2^	Pseudo‐*F*	*p*
${\mathrm{NH}}_{4}^{+}$–N	.050	.030	3.013	**.010**
pH	.063	.033	2.724	**.025**

*Note*: Bolded *p* values indicate statistical significance at the *α* = 0.05 threshold.

## 4 DISCUSSION

Our study investigated, for the first time, the life history, secondary production, and population ecology of a widespread species *R. swinhoei* in a severely eutrophic plateau lake. Our results indicated that *R. swinhoei* had recruitment almost all the year around, with a maximum potential longevity of 2.5 years and a high annual production and *P*/*B* ratio. Moreover, *R. swinhoei* had high density in our study area but showed weak correlations between its population size structure and eutrophication‐related environmental factors, suggesting this snail can thrive in eutrophic conditions.

### 4.1 Life history and secondary production

Species with r‐selected life history traits such as high growth rate, short life span, rapid maturation, and high fertility are expected to be more tolerant to unfavorable environment conditions such as eutrophication than k‐selected species (Barausse et al., 2011; Lin et al., 2014; Mitchell et al., 2018; Moore et al., 2021). R‐strategists tend to devote more energy to reproduction than to growth (Song et al., 2017), which enable them to recover quickly from low population density (Pimm et al., 1988), or to rapidly establish new populations in new freshwater areas (Groom et al., 2006). Few organisms fully conform to “r‐selected” or “k‐selected,” but almost all must reach some compromise between these two extremes (Pianka, 1970; Stubbs, 1977). So, r‐ and K‐strategists is a relative concept, some studies even showed that after the environment changed, the two strategies tended to convert to the other (Song et al., 2017). Our results that *R. swinhoei* exhibited r‐selected life history trend is consistent with our prediction that *R. swinhoei* is a relative r‐strategist. Firstly, it has maximum potential life span (2.5 years), which is much lower than the sympatric endangered k‐selected Caenogastropoda snail *Margarya melanioide* (3.37 years; Song et al., 2017), but similar to the freshwater pulmonates *Planorbarius corneus* (2.1 years) and *Lymnaea stagnalis* (1.3 years; Zotin, 2018). Secondly, *R. swinhoei* can reproduce in almost all year around except winter (December–February), which is similar to *Radix auricularia* in Huai River in China (Li, 1998). Moreover, breeding season of *R. swinhoei* is much longer than many other Pulmonata species, such as *Physa acuta* and *Planorbis planorbis* in Spain (two breeding seasons in a year; González‐Solís & Ruiz, 1996), and Caenogastropoda species *Bellamya aeruginosa* in the East Lake of China (one breeding season in spring per year; Gong et al., 2009). Thirdly, *Radix swinhoei* appears to grow rapidly, because the biomass of *R. swinhoei* population increased significantly in the second month after the three breeding peaks. Fourthly, *R. swinhoei* has fecundity (e.g., *Radix* species could produce 3.53 (±0.19, range: 2–8) egg sacs in each reproductive season in Tibet ponds, and 15.63 (±1.02, range: 2–132) eggs per eggs sac; Yu & Wang, 2013) much higher than many gonochoristic ovoviviparous snails (*Bellamya aeruginosa*: about 15 offspring/brood‐bearing female; *Margarya melanioide*: about 4 offspring/brood‐bearing female; Chen & Song, 1975; Song et al., 2017).

R‐selected species can have significant contribution to the secondary production in eutrophic lakes. Our findings showed high annual secondary production of *R. swinhoei* in Lake Dianchi, which was close to that of *Lymnaea peregra* (147–162 g WW/m^2^) in a British canal (Gaten, 1986), but was higher than that of Caenogastropoda species in other non‐eutrophic shallow lakes in China (e.g., 91.56 g WW/m^2^ for *Bellamya aeruginosa* in Lake East, and 5.00 g WW/m^2^ for *Parafossarulus striatulus* in Lake Biandantang; Gong et al., 2009; Yan et al., 2001). The high secondary production of *R. swinhoei* may be reflective of its adaption to eutrophication, but it does not necessarily represent a healthy nearshore ecosystem in Lake Dianchi. This is because r‐selected species may increase overall benthic secondary production but decrease community complexity (e.g., decreased species diversity and evenness; Dolbeth et al., 2012). Future studies on community‐level secondary production and community composition in nearshore regions of Lake Dianchi are needed to test this idea.

Overall, the r‐selected traits (opportunistic species tendency), reproductive requirements, and relatively high annual production are conducive to the survival and colonization of *R. swinhoei* in the eutrophic water.

### 4.2 Eutrophication‐related drivers of population size structure

A better understanding of response of a species at different ages to eutrophication will facilitate biodiversity conservation in eutrophic ecosystems. In our 1‐year study conducted in the nearshore regions with a high seasonal variation of nutrients (Table 1), pH and ${\mathrm{NH}}_{4}^{+}$–N were identified as most important variables for population size structure of *R. swinhoei* in Lake Dianchi.

Our results showed that pH was positively correlated with small‐sized *R. swinhoei* while being negatively correlated with large individuals. The common explanation for correlation between pH and snail density may be related to weakly acidic habitats, which are harmful to reproduction, hatching, survival, and calcareous shell formation in gastropods, and may lead to high embryonic mortality and low recruitment (Echeverría et al., 2010; Shaw & Mackie, 1989). However, in our study, the pH in surface water of Fubao Bay was basic (pH > 7) throughout the year, which appears to be suitable for *R. swinhoei* survival. The reason why the response to pH differs between juveniles and adults remains unclear but may be related to their difference in tolerance of basic environment.

Our result that small‐sized *R. swinhoei* were correlated negatively with ${\mathrm{NH}}_{4}^{+}$–N suggested that juveniles were sensitive to ammonia nitrogen. Previous studies showed that juveniles of gastropods and mussels were very sensitive to ammonia nitrogen (Hung et al., 2018; Wang, Ingersoll, Greer, et al., 2007; Wang, Ingersoll, Hardesty, et al., 2007). In contrast, we found weak correlations between adults and ${\mathrm{NH}}_{4}^{+}$–N, suggesting that adults are more tolerant of ${\mathrm{NH}}_{4}^{+}$–N. However, we should not interpret the weak association between adults and ${\mathrm{NH}}_{4}^{+}$–N as the ability of the adults to tolerate excessive ${\mathrm{NH}}_{4}^{+}$–N concentration. This is because adults' behavioral and reproductive ability could be negatively affected if they are exposed to excessive ${\mathrm{NH}}_{4}^{+}$–N concentration for a long time, which may further affect population structure and density (Alonso & Camargo, 2009; Hung et al., 2018).

Despite pH and ${\mathrm{NH}}_{4}^{+}$–N identified as important in explaining *R. swinhoei* population structure, we found that the size structure of *R. swinhoei* had a very weak correlation with human‐induced eutrophication factors. This result is different from previous studies on coexisting endangered gastropods *Margarya melanioides* where its population size structure was mainly explained by human‐induced environmental degradation (Song et al., 2013). These contrasting results indicated that *R. swinhoei* are more tolerant and adaptable to eutrophic habitats than *M. melanioides*. One possible explanation why *R. swinhoei* thrive in eutrophic lakes is that it could activate its own antioxidant system to protect against the adverse effects of cyanobacteria toxins (Zhang et al., 2016). Another potential mechanism for *R. swinhoei* to survive in anoxic conditions of eutrophic habitats may be related to its ability to float to surface water to breath air.

This study was mainly based on the relationship between the eutrophication (based on nutrient variations) and the size structure of *R. swinhoei*, which had an enlightening significance for freshwater under the increasing eutrophication. However, the study of only one trophic level waterbody may limit the gradient difference of environmental factors, and it may be necessary to explore the ecology of different waterbodies (such as different eutrophication states, pH range, etc.) simultaneously in the future to strengthen the universality of the research conclusions.

### 4.3 Other drivers of population size structure and density

Other drivers not considered in this study could also influence the abundance and population structure of *R. swinhoei*. Coverage of aquatic plants may be important because periphyton on aquatic plants and/or aquatic plants themselves serve as food sources for *R. swinhoei*. Additionally, wind‐induced waves might influence geographic distribution of *R. swinhoei* and its population structure in nearshore regions of Lake Dianchi by the haphazard transport of different size of *R. swinhoei* (Jin et al., 2022). Finally, fecundity and reproduction potential should be considered in the future studies of quantifying long‐term population dynamics of *R. swinhoei*.

## 5 CONCLUSIONS

Our finding suggested that *R. swinhoei* is a r‐strategist in Fubao Bay of Lake Dianchi, with short maximum potential longevity (2.5 years), three recruitment periods, and high annual secondary production (137.19 g WW/m^2^). Our results showed that two eutrophication‐related factors (e.g., pH and ${\mathrm{NH}}_{4}^{+}$–N) were the important drivers, and small individuals (shell length from 2.00 to 9.99 mm) were positively correlated with pH but negatively correlated with ${\mathrm{NH}}_{4}^{+}$–N. Despite the importance of those variables for *R. swinhoei*, eutrophication‐related factors explained little variation in its population size structure, suggesting that *R. swinhoei* thrive in eutrophic environment. Our study, the first to address life history, secondary production, and population ecology of *R. swinhoei*, emphasized the potential importance of widespread species in eutrophic ecosystems. Our findings have implication for other widespread species in eutrophic environment, and generality should be tested in other eutrophic ecosystems.

## AUTHOR CONTRIBUTIONS

**Junqian Zhang:** Conceptualization (lead); investigation (lead); methodology (lead); writing – original draft preparation (lead); writing – review and editing (equal); formal analysis (lead). **Zhuoyan Song:** Methodology (equal); writing – review and editing (equal). **Zhengfei Li:** Data curation (equal); formal analysis (equal); methodology (equal); validation (equal); writing – review and editing (equal). **Jiali Yang:** Visualization (supporting); writing – review and editing (supporting). **Zhicai Xie:** Conceptualization (lead); writing – review and editing (equal).

## Data Availability

Relevant population data and environmental data input files: Dryad doi: 10.5061/dryad.t76hdr836.
